# Current Trends and Perspectives of Polymers in Batteries

**DOI:** 10.1021/acs.macromol.3c01971

**Published:** 2024-03-19

**Authors:** David Mecerreyes, Nerea Casado, Irune Villaluenga, Maria Forsyth

**Affiliations:** † POLYMAT, University of the Basque Country UPV/EHU, Avenida Tolosa 72, Donostia-San Sebastián 20018, Spain; ‡ IKERBASQUE, Basque Foundation for Science, Bilbao 48011, Spain; § Institute for Frontier Materials, Deakin University, Burwood, VIC 3125, Australia

## Abstract

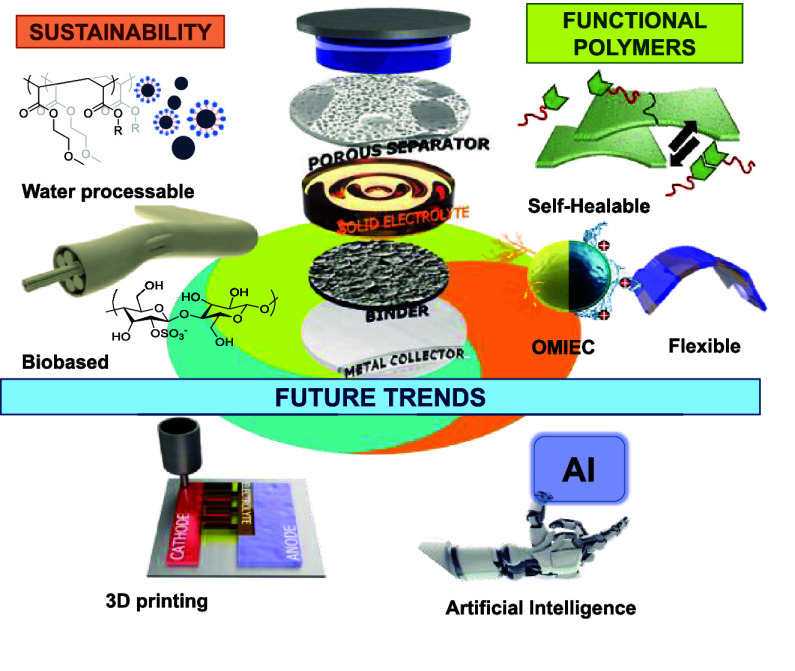

This Perspective aims to present the current status and future opportunities for polymer science in battery technologies. Polymers play a crucial role in improving the performance of the ubiquitous lithium ion battery. But they will be even more important for the development of sustainable and versatile post-lithium battery technologies, in particular solid-state batteries. In this article, we identify the trends in the design and development of polymers for battery applications including binders for electrodes, porous separators, solid electrolytes, or redox-active electrode materials. These trends will be illustrated using a selection of recent polymer developments including new ionic polymers, biobased polymers, self-healing polymers, mixed-ionic electronic conducting polymers, inorganic–polymer composites, or redox polymers to give some examples. Finally, the future needs, opportunities, and directions of the field will be highlighted.

## 1 Introduction

Batteries as electrochemical energy storage devices are present in our daily life everywhere from watches to computers or electric vehicles. Most commercial batteries nowadays are based on lithium ion battery chemistry (LIB), and this discovery was recognized with the Nobel Prize award in 2019.^1^ Today, in every commercial lithium ion battery, there are polymers present as inactive components such as binders for the electrodes or porous separators for embedding the liquid electrolyte.^2^ Thus, well-known polymers such as poly(vinylidene fluoride) (PVDF) binders and polyolefin porous separators are used to improve the electrochemical performance and stability of the batteries.

Furthermore, functional polymers play an active and important role in the development of post-Li ion batteries. In particular, ion conducting polymer electrolytes are key for the development of solid-state battery technologies, which show benefits mostly related to safety, flammability, and energy density of the batteries. An example is found in the solid-state lithium metal batteries, which make use of poly(ethylene oxide) (PEO) as a matrix of an ion conducting solid electrolyte. In this sense, the improvement of the performance of PEO-based polymer electrolytes and the search of new polymers are a very active topic of research by the industrial and scientific community.^3,4^ This is mostly due to the current development of high-energy battery technologies based on lithium metal and high-voltage cathodes but also future development of different battery chemistries involving alternative metals to lithium such as sodium, potassium, or divalent ions such as zinc, magnesium, or calcium.^5^

Another concern of Li ion batteries is found in the use of toxic and scarce metals (e.g., cobalt, nickel, and manganese) as electrode materials. These concerns range from the scarcity of some of these elements and their topo-geographical limited harvesting to the footprint of their energy-intensive mining and processing. Organic electrode materials (OEMs) have gained much attention as sustainable alternative materials for emerging battery technologies.^6,7^ This is mainly due to the abundance of their elements, their energy-efficient processing, and the ease of their modification by organic chemistry. Among the organic electrode materials, redox-active polymers are a very active topic of current research due to the interest in new organic battery technologies.^8^ Some decades ago, already unsuccessful industrial attempts took place using redox-active conducting polymers or radical polymers as electrodes in batteries/supercapacitors. However, there is now a significant resurgence in interest due to the emergence of new battery technologies such as paper batteries, flexible batteries, metal–polymer, polymer–air, or organic redox flow cells where redox polymers play an active pivotal role.^9^

The goal of this Perspective is to summarize important issues in the use of polymers for lithium ions as well as emerging battery technologies. This will include the current developments of polymer binders, porous separators, polymer electrolytes, and redox polymers. The aim is to highlight the different polymer targets that are being investigated and consider new aspects such as sustainability.

## 2 Polymers As Binders of Electrodes

The polymer binder is responsible for maintaining the contact and structural integrity of the electrodes by connecting the redox-active material with conductive agents and current collectors (Al or Cu foil). Although binders only occupy 2–5% of the weight and about 1% of the price of a commercial Li ion battery, they play a crucial role for the battery performance, cycle life, safety, and sustainability. Without the binder, the active materials will lose contact with the current collector, resulting in capacity loss and an eventual short circuit. Thus, binders should possess several properties to ensure a good performance: (i) high thermal, chemical, and electrochemical stability, (ii) flexibility, (iii) insolubility in electrolyte, (iv) strong adhesion, (v) the ability to form uniform electrodes, (vi) cost-effectiveness, and (vii) environmental friendliness.^10^ Furthermore, although there are some generalities, the requirements of the binder are specific to each electrode material.

For the processing of inorganic cathodes in Li ion batteries, poly(vinylidene difluoride) (PVDF) and its copolymers are the most commonly used binders due to their good electrochemical stability and strong adhesion, together with the good compatibility and absorption of the electrolyte that promotes lithium transport to the active material surface. However, PVDF has a high fluorine content and requires the usage of the toxic and expensive organic solvent *N*-methyl-2-pyrrolidone (NMP) for the electrode processing and elevated temperatures for the drying step, making the cathode manufacturing process environmentally unfriendly. As an alternative to PVDF, low-fluorine-content poly(ionic liquids) or anionic single-ion lithium conducting polymers are demonstrating enhanced cycling stability and capacity values in Li–air and solid-state batteries.^11−13^ As a recent example, pyrrolidinium-based poly(ionic liquid)s were reported as cathode binders in NMC cathodes which showed improved capacity values, rate performance, and cycling stability,^14^ In particular, PDADMA–CFSO showed a cell capacity increase of 26% at 5 C when compared to PVDF.

Furthermore, it is necessary to develop cost-effective and eco-friendly binders as alternatives to PVDF. For this reason, the use of biopolymers and water-processable polymeric binders is increasingly investigated as a more sustainable solution.^15,16^ However, the water processing of the cathodes usually leads to a worse battery performance. Challenges in the water processing of cathode materials include crack formation, substrate degradation of active materials due to the contact with water, corrosion of aluminum current collector, and leaching of lithium ions decreasing the capacity of cathodes. Among the most successful aqueous binders for cathodes are PVDF latexes or carboxymethyl cellulose (CMC) coupled with other biopolymers such as alginate or chitosan, rubber latexes or poly(acrylic acid), and poly(ethylene oxide) (PEO). However, the water processing of new high-voltage cathodes such as NMC-type materials is still challenging due to their low stability in water. Here, some recent successes have been reported with NMC_811_ with the use of ionic polymers such as anionic carrageenan biopolymers or water-soluble phosphate–poly(ionic liquid)s.^17^

On the other hand, alternative anode materials to graphite are also being sought in order to achieve high-energy-density batteries. Here, silicon-based anodes are ideal candidates due to their high theoretical specific capacity of pure silicon 4200 mAh g^–1^. However, during the lithiation/delithiation process silicon can suffer a volume expansion of up to 400%, causing rapid capacity decay and safety concerns.^18^ Thus, binders which help to accommodate the volume and chemical changes of silicon anodes, with for instance elastomeric and self-healing properties, have been searched for. These functional binders should support the retention of the original structure of the electrode and maintain the contact integrity between the Si active material, conductive additive, and current collector.^19^ In this sense, elastomeric and self-healing polymers that can develop strong intermolecular interaction through hydrogen bonding as well as dynamic ionic and covalent bonds are being investigated.^20^ As a pioneering example, Bao and co-workers developed a self-healing polymer based on a polyurea polymer functionalized with poly(ethylene glycol) (PEG) groups, where the hydrogen bonds of ureas enabled the adhesion to Si surface and provided self-healing properties, while the PEG units promoted the Li conduction within the binder. They achieved a high discharge capacity of ≈2600 mAh g^–1^ and a capacity retention of 80% after ≈150 cycles at 0.5 C.^21^

Besides the conventional binders currently used in cathodes and anodes, multifunctional binders having intrinsic ionic and electronic conductivity, which improve the interaction between the electrode and electrolyte, are being developed. The use of electronic conductive binders can show advantages in both the increased loading of active materials and the rate performance of the batteries. Thus, conducting polymers such as PEDOT:PSS, PPy, and PANI have been investigated as binders in cathodes for the Li ion, Li–S, and LMBs, as well as in silicon anodes.^22^ Here, mixed ionic–electronic conducting binders have emerged as promising materials to reduce the carbon content of cathodes as well as improve the rate capability and capacity values in batteries.^23^ In one recent example, mixed ionic–electronic conducting binders based on PEDOT:PSS and organic ionic plastic crystal facilitated a solid-state battery containing a LFP cathode without any carbon additive. Thus, the mixed conducing binder replaced both the conventional binder and carbon additives in the solid-state Li/LiFePO_4_ cell, obtaining an enhanced discharge capacity (157 mAh g^–1^ at C/10) and improved rate capability, in comparison to a solid-state cathode formulation using an ionic conducting polymeric binder and carbon additive.^24^ The specific capacity of 145.5 mAh g^–1^ at a C/2 rate was achieved with a capacity retention of 99.7% after 500 cycles. Another type of multifunctional polymer applied as binder is materials with inherent redox activity. The redox-active site of the binders can facilitate an increase in capacity by redox-mediating the electrochemical process of the electrode. Some examples of this strategy involve the introduction of TEMPO containing polymers as binders or the use of supramolecular polyimides.^25,26^

**Figure 1:**
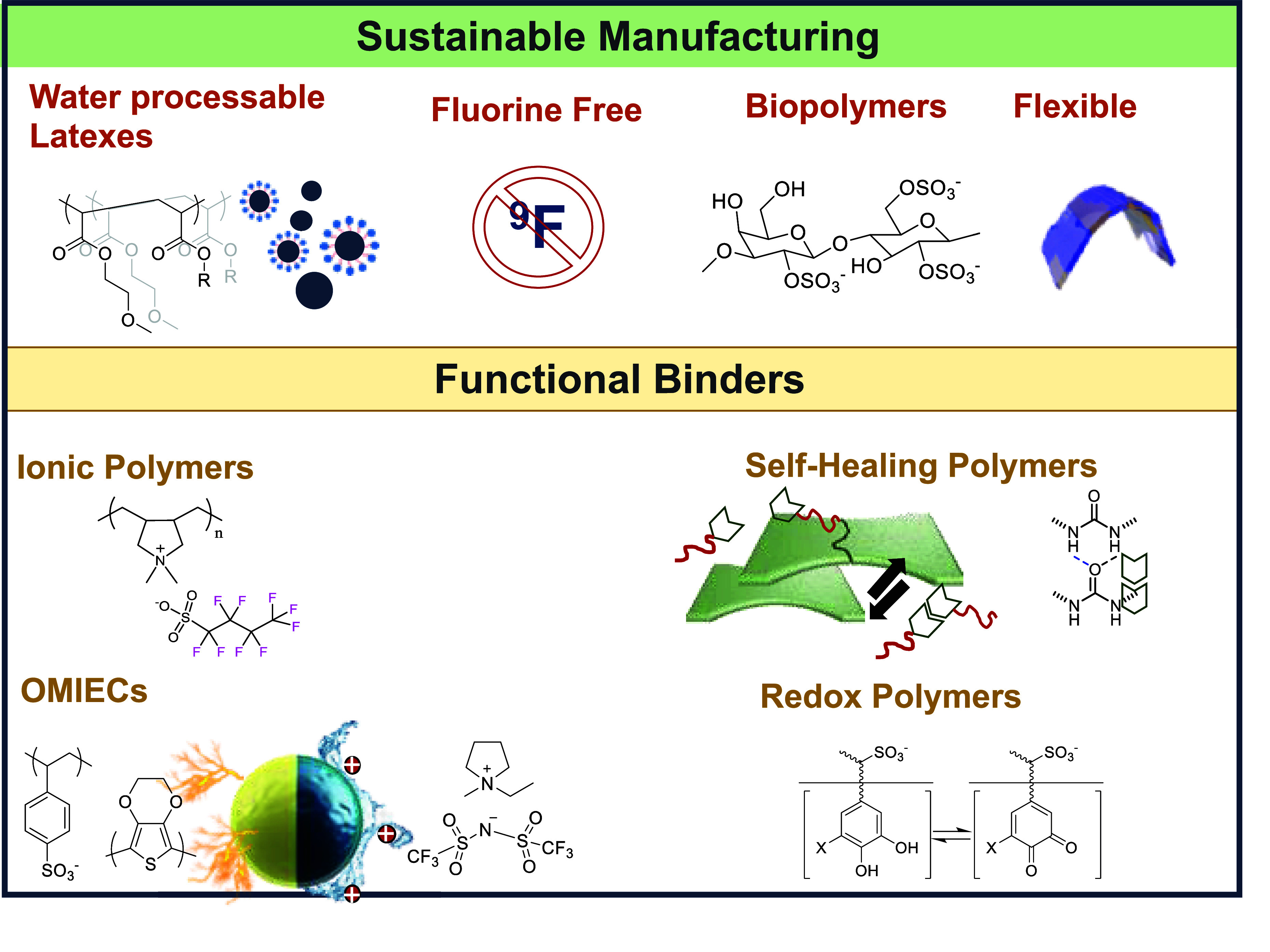
Representation of current trends in polymers investigated as binders in battery electrodes.

## 3 Polymers As Separator Electrolyte

### 3.1 Porous Separators

**Figure 2:**
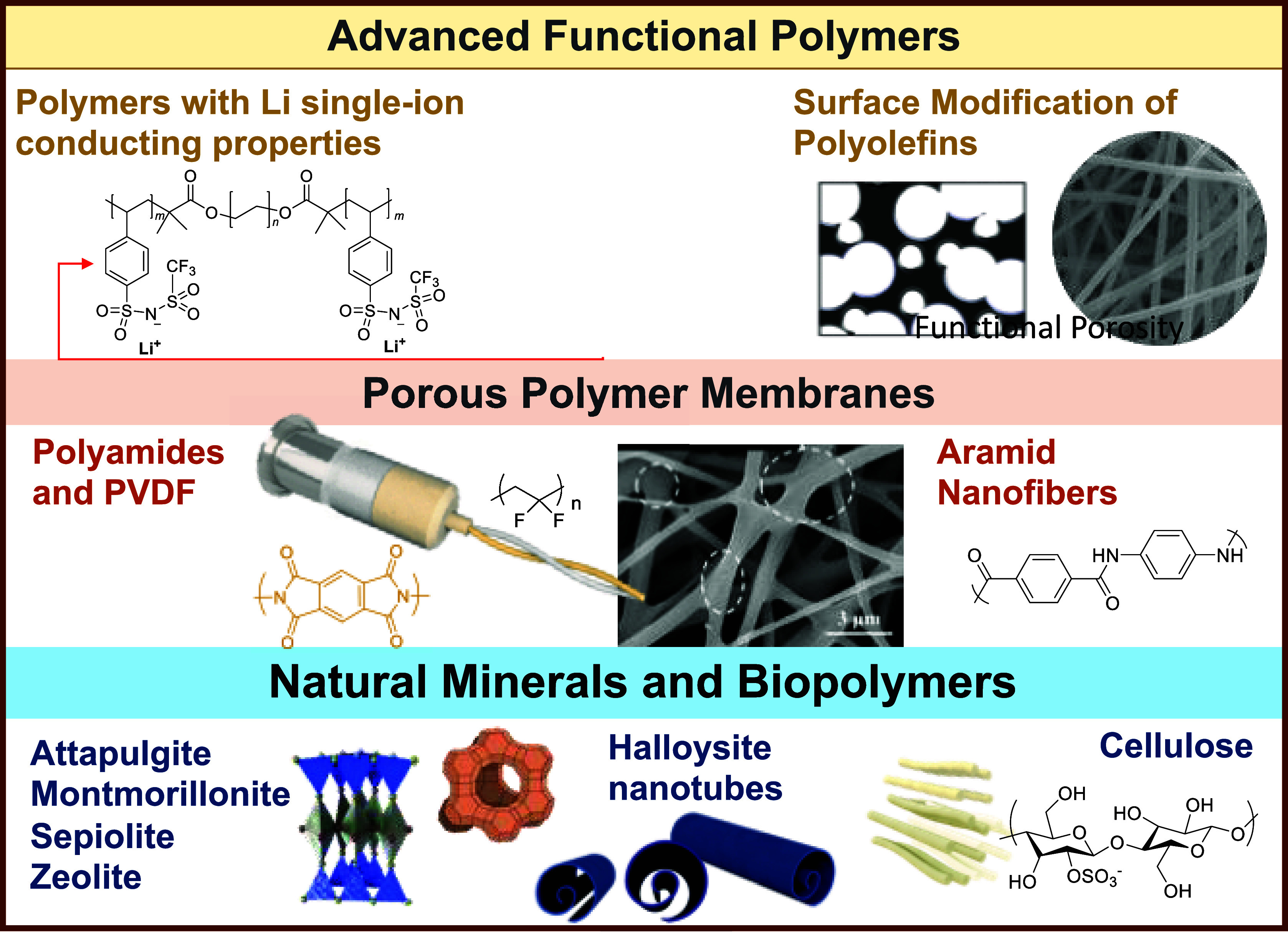
Representation of current trends in polymers investigated as porous separators.

The current porous separator market is dominated by polyolefin-based membranes. However, their limited wettability with new electrolytes, thermal shrinkage, and thermal meltdown properties limit the safety and high-power applications of lithium ion batteries. A current trend is to modify chemically or physically the surface of the polyolefin separator in order to increase its electrolyte compatibility.^27^

In recent years, most efforts have been made to develop new separators in order to improve commercial polyolefin separators. Poly(vinylidene fluoride) (PVDF) and its copolymers are one of the most investigated alternatives to polyolefins due to good mechanical strength, good wettability, chemical inertness, and thermal stability. Furthermore, their porosity and pore size can be tuned by using different routes.^28^ For instance, Widiyandari et al. investigated the use of PVDF nanofibers obtained by electrospinning with varying porosity, morphological, and thermal properties.^29^ The mechanical and thermal stability properties can be enhanced when PVDF is blended with other polymers (PI, PMMA, PEO, etc.). For instance, Cai et al. recently blended PVDF–HFP with polyimide (PI) to improve the mechanical strength of the electrospun separator. The authors reported that PVDF–HFP/PI bicomponent electrospun separators with cross-linked structures present improved mechanical strength, electrolyte uptake, ionic conductivity, and thermal stability.^28^ The addition of nanofillers (SiO_2_, Al_2_O_3_, TiO_2_, Sb_2_O_3_, etc.) into the polymer matrix is a common strategy to improve the porous separator properties such as electrolyte uptake and mechanical and thermal stability.

It is also worth mentioning the use of high-performance aramid nanofibers based on poly(*p*-phenylene terephthalamide) (PPTA) as porous separator.^30^ Thus, Patel et al. developed a pure nanofiber membrane-based separator, which showed a high Young’s modulus (8.8 GPa), high tensile strength (253 MPa), inherent flame retardancy, and outstanding thermal resistance (*T*_d_ of 509.6 °C).^31,32^ However, it exhibits an unexpected extremely poor battery cycling performance, probably due to poor porosity and electrolyte uptake. Several approaches have been reported to improve the porous structure of the nanofiber membrane, including regulation of the morphologies, porous structure control (introduction of nanoparticles and wet stretching), and optimization of drying methods of the membrane.^33^ Another class of high-temperature-resistant polymers that have been widely developed as porous separators is the polyimides (PI). Polyimide separators have remarkable high heat resistance, flame retardancy, chemical resistance, and good wettability and battery performance; however, the manufacturing process is complicated and expensive, which hinders its industrial production.^34^

In the past few years, biobased sustainable materials and low-cost materials such as natural minerals and biopolymers have attracted widespread attention in separators. For instance, halloysite nanotubes, attapulgite, sepiolite, montmorillonite, zeolite, and diatomite are some examples of inorganic materials based on natural minerals used in polymer nanocomposites.^35^ They present excellent thermal/chemical stability and mechanical properties with unique micro- and nanopore structures. Moreover, they can improve the thermal shrinkage and poor mechanical strength of the current polyolefin separator. On the other hand, the most studied biopolymers are those based on cellulose and its fibrous derivatives such as nanocellulose.^36^ They hold great potential to be implemented as separators due to flexibility, inherent porous structure, biodegradability, low cell resistance, and high electrolyte uptake. Nevertheless, currently there are not cost-effective routes for scale-up of the manufacture of biopolymers.

Beyond the conventional inert porous separators, new polymers with different functionalities, such as the presence of lithium ions within the polymer and self-healing moieties, are being developed. For instance, Balsara and co-workers showed a nanostructured ionic separator based on a triblock copolymer, poly(styrenesulfonyllithium(trifluoromethylsulfonyl)imide)-*b*-polyethylene-*b*-poly(styrenesulfonyllithium (trifluoromethylsulfonyl)imide) (PSLiTFSI-*b*-PE-*b*-PSLiTFSI). This copolymer self-assembles into nanophase-separated lamellae morphology and has lithium single-ion conducting properties, avoiding the concentration polarization and thus providing a potential solution to enable fast charging and discharging of lithium ion batteries.^37^

In summary, different materials and fabrication methods are being investigated in the search of functional porous separators with improved properties such as electrolyte uptake, flame retardancy, or thermal stability. Understanding of the relationship between the chemical nature of the polymer, its functionality, its porous morphology, electrolyte uptake, and performance is essential for the improvement of the porous separators currently used in batteries. The future development of these separators will be marked by the need of facilitating the recycling of battery wastes as well as the development of sustainable biobased porous separators.

### 3.2 Polymer Electrolytes for Solid-State Batteries

As mentioned in the Introduction, PEO although discovered in the 1970s is still the material of choice as matrix for polymer electrolyte used in lithium metal solid-state batteries.^38^ A great deal of research was devoted in the past decades to formulate PEO-based electrolytes in the forms of blends, nanocomposites, or block copolymers to improve its properties. PEO shows a great ability to dissolve lithium salt and shows very high values of ionic conductivity (up to 10^–3^ S/cm at 70 °C). However, PEO-based electrolytes have several limitations. The first one is related to its limited electrochemical stability window to operate batteries based on high-voltage cathode materials, and the second one is its low lithium transference number which affects battery operation.^3^ However, in the past years, other aspects such as safety, flammability, sustainability, recyclability, self-healing, or biodegradability of the polymer electrolytes are also gaining importance. The next paragraphs will show examples of these trends through some recent works on polymer electrolytes beyond PEO.

One important benefit of the solid electrolytes is associated with the safety of the batteries, for instance, the flame retardancy, which is of paramount importance in applications such as electric vehicles or personal electronics. Here, although PEO polymer electrolytes behave well compared to organic flammable electrolytes, the flame retardancy aspects of the PEO polymer electrolyte can be improved. Typical strategies to improve flame retardancy have been the use of additives such as ionic liquids or other flame retardants. However, in the past years the development of polymers with intrinsic flame-retardancy properties such as poly(phosphoesters) or phosphonate functional polymethacrylates have been shown as alternative polymer electrolytes.^39,40^

In the past decade, a very popular family of polymers used as polymer electrolytes are the ionic conducting poly(ionic liquid)s. Among them, the poly(diallyldimethylammonium) (PDADMA) polycation with counteranions such as sulfonamides (FSI or TFSI) are being extensively investigated as a polymer electrolyte matrix in combination with ionic liquid electrolytes^41^ or high lithium salt concentrations.^42^ These poly(ionic liquid) polymer electrolytes show high ionic conductivity values and an electrochemical stability window coupled with high lithium transference numbers, thus improving some of the properties of traditional PEO-based solid electrolytes. This allows them to be used with high-voltage cathodes such as the NMC 811 or LMNO. Lithium metal batteries running at 25 °C with high performance have recently been demonstrated.^43−45^ Future improvements of the PDADMA polymers in terms of mechanical stability or miscibility with new ionic liquid electrolytes are expected by developing new copolymerization strategies. Recent examples of the copolymerization approach to improve the mechanical properties of poly(ionic liquid)-based polymer electrolytes while retaining their favorable ionic conductivity and lithium transport number are the RAFT polymerized diblock and triblock polymers; when combined with LiFSI and an ionic liquid, the resulting polymer electrolyte was demonstrated to give excellent performance in Li metal batteries.^43,44^ Interestingly, PDADMA-based polymer electrolytes have shown great versatility and performance in other battery chemistries such as sodium metal batteries.^46,47^

Another property of PEO polymer electrolytes that is detrimental to optimum performance in batteries is their relatively low lithium transference number, which is usually *t*_Li_ < 0.25. Hence, in recent past years research has been focused on the use of carbonyl-containing polymers such as polycarbonates or polyesters which may show lithium transference numbers in the order of 0.5 while still showing good ionic conductivity values.^48^ To further push the lithium transference number close to unity, lithium single-ion polymer electrolytes have received great interest in the past few years. Among them, the sulfonamide anionic polymers with styrene or methacrylic backbones have been the most popular in the form of block copolymers, gels, or polymer blends to tune material properties.^49^ However, the lithium single-ion conducting homopolymers still show a limited ionic conductivity, due mostly to the restricted mobility of ions arising from the high glass transition of the lithium sulfonamide polymers. To circumvent this issue, new low-*T*_g_ single-ion conductors such as lithium borate containing polymers have recently been developed which show ionic conductivity values close to the PEO polymer electrolytes while showing lithium transference numbers close to 0.9.^50^

So far, most of the efforts were devoted to develop polymers with improved electrochemical properties (ionic conductivity, electrochemical window, and lithium transference numbers). However, there are aspects related to sustainability, circularity, or carbon neutrality of the polymers used as polymer electrolytes that are increasingly important. Recycling of the batteries is becoming an issue, in particular, with the expected increase of battery production in the next years due to the emergence of multiple gigafactories. For this reason, aspects like using polymers which may be biosourced or biodegradable or the use of CO_2_ for their synthesis are becoming more and more important. As an example of the use of biosourced polymers, cellulose nanofibers are becoming a popular reinforcement of composite polymer electrolytes as reviewed very recently by Lizundia et al.^51^ In this line, several recent works are related to the design of polymer electrolytes synthesized using CO_2_, for instance, the biodegradable aliphatic polycarbonate solid electrolytes pioneered by Tominaga et al. which can be synthesized by the copolymerization of CO_2_ and epoxides.^52^ More recently, Detrembleur et al. showed the development of polycarbonate polymer electrolytes by polycondensation of CO_2_-sourced bis(α-alkylidene carbonate) monomers.^53^ In another example, Williams et al. recently reported the synthesis of optimized polycarbonate block copolymers for solid-state batteries.^54^

New polymer electrolytes are also being designed taking into account aspects like recyclability. As one example, Sardon et al. recently showed the use of the chemical recycling–upcycling concept of plastics to develop polymer electrolytes. As an example, special carbonate diols were obtained by depolymerization of bisphenol A polycarbonate waste which was used for the development of polymer electrolytes for lithium ion batteries.^55^ In another example, Bella and co-workers showed a separator based on dynamic covalent adaptable networks or vitrimers that allow its reprocessing. Furthermore, dynamic chemistry brings to the lithium ion battery additional self-healing and self-repair properties.^56^

**Figure 3:**
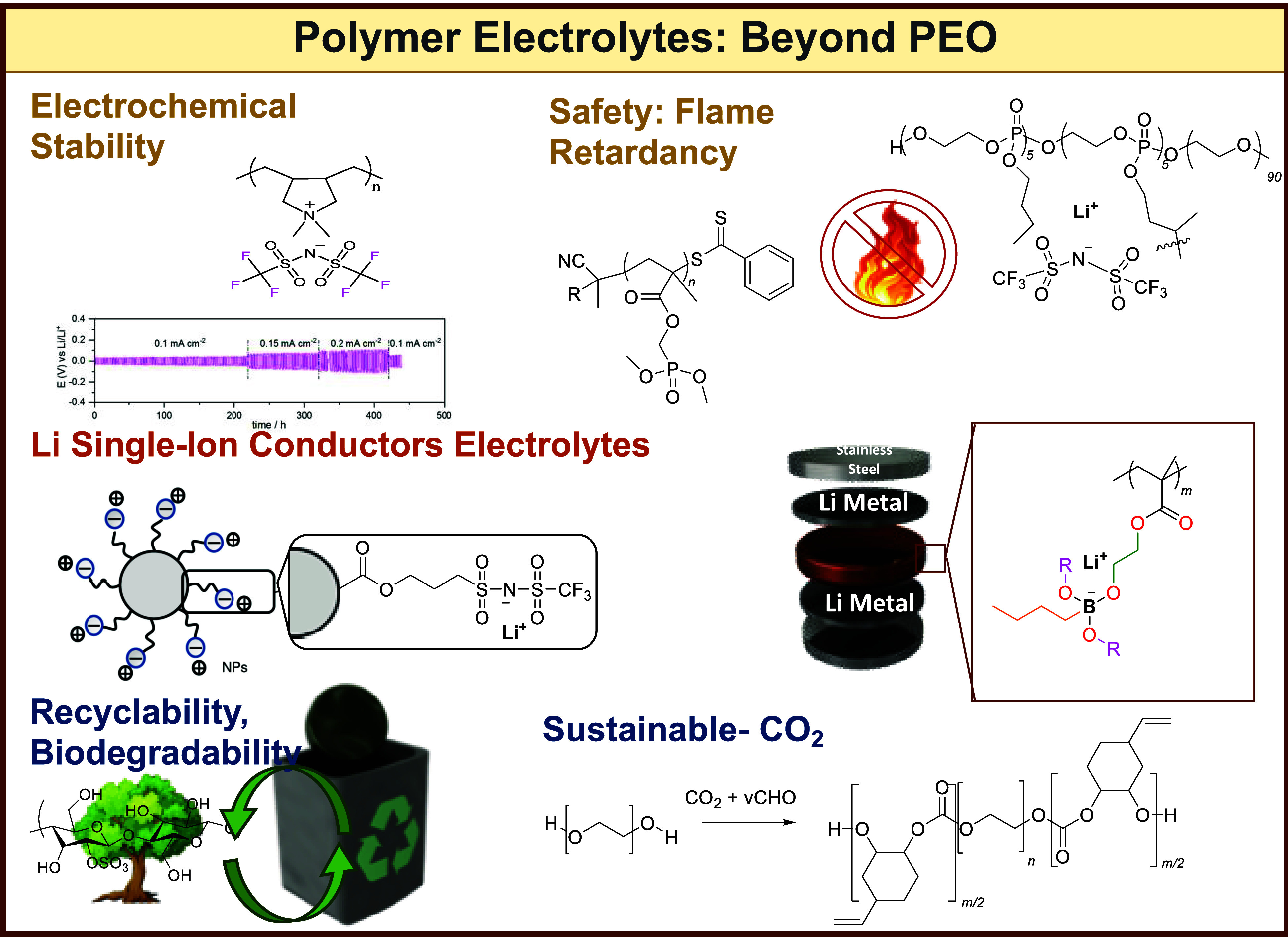
Representation of current trends in polymers investigated as polymer electrolytes.

### 3.3 Inorganic–Polymer Composite Electrolytes or Hybrid Solid Electrolytes

Inorganic conductors such as oxide-based solid electrolytes (garnet-type LLZTO (Li_7_La_3_Zr_1.4_Ta_0.6_O_12_), perovskite-type LLTO (Li_3*x*_La_2/3–*x*_TiO_3_), etc.), sulfide-based solid electrolytes (Li_2_S–P_2_S_5_, Li_6_PS_5_Cl, etc.), and halide-based electrolytes (Li_3_InCl_6_, etc.) are single-ion conductors with high shear moduli and high ionic conductivity at room temperature.^57^ On the other hand, the polymer electrolytes present inherent problems such as insufficient ionic conductivity at room temperature. Attempts to overcome the limitations of both materials have led to great interest in inorganic–polymer composite electrolytes.

An ideal composite electrolyte should be able to (1) form strong interactions between inorganic particles to maintain adhesion over cycling, (2) offer strong adhesion toward electrodes to prevent high interface resistances, (3) provide a continuous conductive network within the composite electrolyte, (4) exhibit sufficiently high yield stress to accommodate volume changes during battery cycling without breaking, (5) be electrochemically and chemically stable, (6) be compatible with high-voltage cathodes and Li metal and (7) with current battery manufacturing lines, and finally (8) be competitive at low prices. The composite electrolytes are usually classified in inorganic high rich content (>50 vol % of the inorganic electrolyte) and polymer high rich content (<50 vol % of the inorganic electrolyte). The volume of the inorganic electrolyte will determine the morphology and the ion transport processes of the resulting composite electrolyte.^58−60^

**Figure 4:**
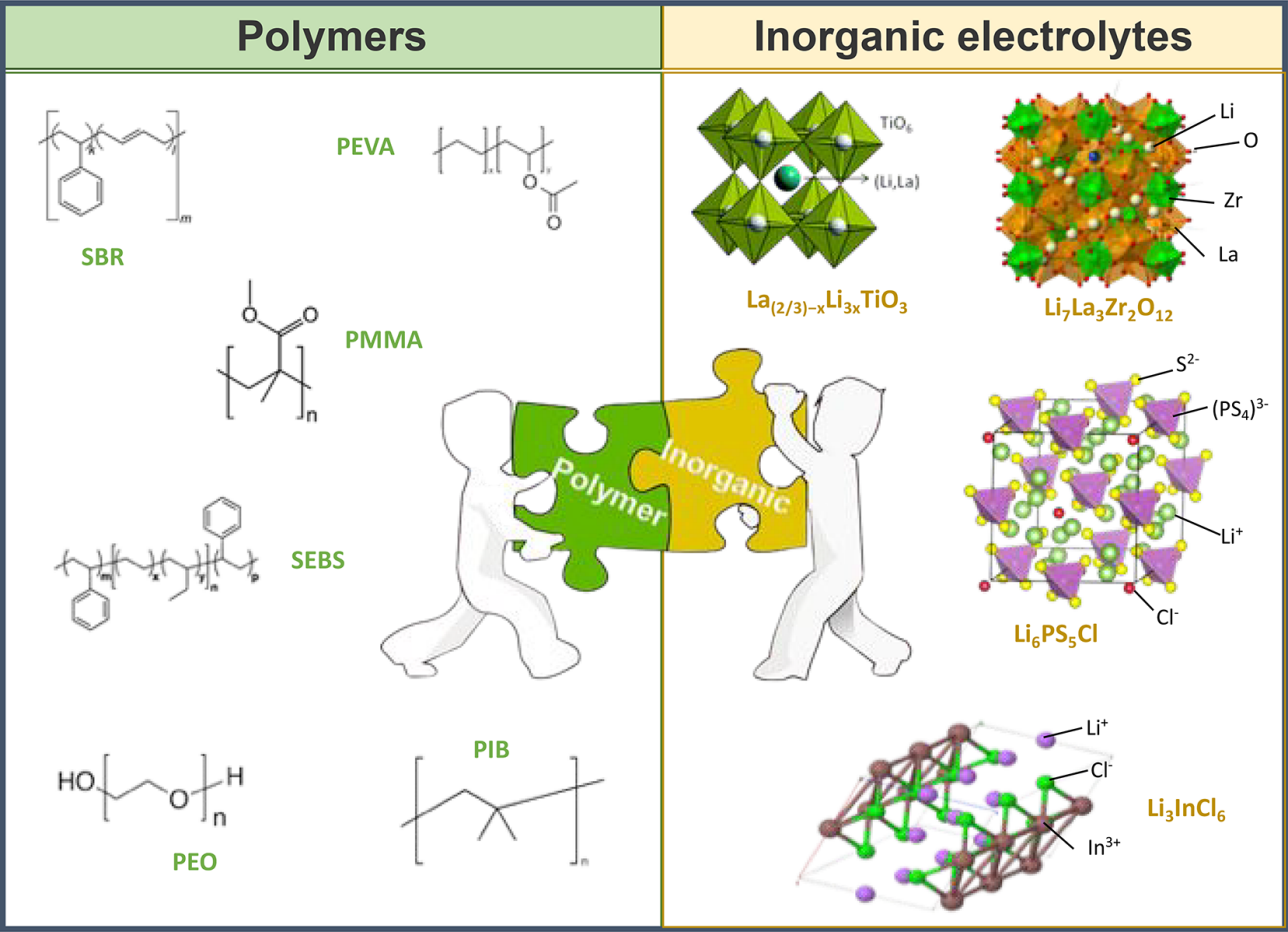
Representation of types of hybrid composite electrolytes investigated as solid electrolytes.

In inorganic-rich composite electrolytes, the polymer acts as a “glue” for the inorganic particles and provides intimate adhesion to the electrodes and the active materials during cycling, while the inorganic particles impart a high modulus and provide highly conductive domains raising the overall electrolyte conductivity notably at room temperature. The transport process can take place in the inorganic phase, along the interface, or across both phases. The most common synthetic methods include mixing in a solvent, followed by solvent-casting and hot-pressing. The selection of the solvent is a key parameter to avoid the degradation of chemical structure of the inorganic electrolyte and, therefore, the transport properties in inorganic high rich composite electrolytes. The solvent must be compatible with the polymer but at the same time be inert for the inorganic electrolyte. Given that the inorganic electrolytes are unstable in polar solvents (such as NMP, DMF, and ACN), this limits the choice of the polymer. For example, Tan et al.^61^ observed that the solvent with high dielectric constant leads to the decomposition of the sulfide electrolyte (Li_7_P_3_S_11_), generating undesired products and very low ionic conductivities. The choice of the polymer is crucial, although it is a challenge to find adhesive polymers that can be soluble into nonpolar solvents. The current strategy is the grafting of polar functional groups in nonpolar polymers. Riphaus et al. investigated the impact of different polymers in sulfide-based composite electrolytes on the morphology, processing, porosity, and ionic conductivities.^62^ The higher the molecular weight, the less binder that is needed in order to obtain freestanding composite electrolytes. Combination of a nonpolar polymer backbone with a moderate amount of grafted polar functional groups was found to provide the composite electrolyte with the best ionic conductivities. However, bulky side groups seem to hinder the proper adhesion of the polymer to the inorganic particles. This leads to the need for higher polymer amounts to obtain mechanically stable composite electrolytes. Lee et al. developed a series of carboxylic acid-functionalized styrene butadiene-*block*-copolymer synthesized by thiol–ene click reactions.^63^ The polymer with 10% carboxylic acid groups is still soluble in *p*-xylene, which is compatible with the sulfide-based electrolyte. This functional elastomeric binder provides a uniform electrode without degradation of the chemical structure of the sulfide electrolyte while improving the electrode adhesion 1.4 times compared with that of commercial PVdF-based electrodes.

In inorganic-rich composite electrolytes based on oxide solid electrolytes, a simple combination of a polymer matrix and an inorganic electrolyte does not provide high performance. This is because a percolating network cannot be formed by inorganic particle contact, which results in low conductivity values at room temperature.^62^ To overcome this limitation, 3D printed templates, with both aligned and random distribution of oxide-based electrolyte nanofibers, were developed by several research groups by electrospinning. The flexibility is achieved by the infiltration of polymer or polymer electrolytes (PEO, PEO+LiTFSI, etc.) into the porosity within the inorganic nanofibers. The ionic conductivities of the composites are in the order of 10^–4^ S/cm at room temperature. When polymer electrolytes are used with inorganic electrolytes, the ion transport processes can happen across both phases. However, the bicontinuous or 3D interconnected morphologies provide very fast lithium ion diffusion in the inorganic phase, and thus, the polymer electrolyte acts merely as a binder without any significant contribution to the ionic conductivity.

In polymer-rich composite electrolytes, the ion transport processes can happen in the polymeric phase, or along the interface/interphase, or across both phases.^64−66^ Some reported polymer-rich composite electrolytes show decreased ionic conductivities compared with the inorganic electrolyte but improved ionic conductivities compared to their pure polymer electrolyte. As one example, Lechartier et al. developed a hybrid solid electrolyte formed by a poly(ethylene glycol)-type single-ion polymer network and a ceramic garnet type (LLZO) with a very high lithium conductivity (1.4 × 10^–4^ S/cm at 25 °C).^67^ This approach showed excellent dispersion of the LLZO nanoparticles within the gel polymer network with up to 40 wt % ceramic content. For low LLZO compositions, the increase in the ionic conductivity is attributed to the decreased crystallinity of the polymer matrix caused by the presence of the conductive filler. Up to 40 wt % of LLZO, the percolation pathways are established in the inorganic particles, and the ionic transport mechanism happens in the inorganic phase. However, some other studies show a decrease of the ionic conductivities of the composite compared to the pure polymer electrolyte.^68^ This is probably because of the kinetically slow Li^+^ ion transfer across the polymer–inorganic interfaces, leading to an increased tortuosity of the lithium ion pathways in the polymer matrix by the inorganic particles. In polymer-rich composite electrolytes, one of the major challenges in combining inorganic and polymer electrolytes is the transfer resistance between both materials. A typical example is the composite electrolytes composed of inorganic electrolytes and PEO–LiTFSI. The ionic conductivity is low at room temperature because the inorganic particles are embedded and are isolated from participating in the overall ionic conductivity of the composite electrolytes.

To conclude this section, despite the extensive research, many fundamental questions remain unanswered in composite electrolytes such as the morphology control of the composites or the mechanisms of ion conduction through the different phases and the role of the inorganic–organic interphases. In addition, the incompatibility of most of the hybrids with lithium metal due to the interaction of inorganic materials makes the fabrication and integration in cells at the pilot line challenging. For this reason, interlayers and robust solid electrolyte interfaces are needed nowadays in the case of lithium metal batteries. Besides these challenges, the area of composite hybrid electrolytes is growing, and new material combinations and engineering solutions are being developed to circumvent these issues.

### 3.4 Redox Polymers toward New Battery Technologies

As discussed in the Introduction, redox polymers that can be reversibly oxidized and reduced are gaining much attention as sustainable electrode materials. The main advantages are that redox polymers can be chemically tuned and biobased, thus enabling materials for new battery technologies such as paper batteries, organic redox flow batteries, polymer–air batteries, or flexible organic batteries. The core challenges are still the cycling stability and reliability compared to the dominant inorganic redox materials in batteries. In the next paragraphs, we will discuss some current trends in the design and applications of new redox polymers.

In terms of performance, redox polymers have demonstrated higher capacities and performance at high rates as compared to inorganic electrode materials, whereas they present issues such as low electrode loading, battery self-discharge, and low cyclability. For example, the intensively studied anthraquinone and catechol-based polymers are able to deliver capacities of up to 200 and 360 mAh g^–1^, respectively, while typical inorganic cathode materials usually deliver capacities of around 140 mAh g^–1^. However, despite high theoretical capacities, only a few materials have been reported to display high redox potentials of 3.5 V (vs Li/Li^+^) such as the TEMPO-based electrodes pioneered by the group of Nishide and Oyaizu,^69^ necessary for high-power batteries. Thus, one of the current trends is to develop redox polymers that can be used as high-voltage cathodes (close to 4 V). As an illustrative example, Esser and co-workers developed phenothiazine-based redox polymers.^70^

The high electrochemical performance of redox polymers is associated with the use of low mass-loading electrodes with high carbon content (up to 50 wt %), in contrast to inorganic materials (5 wt %). This fact limits the application of redox polymers in commercial batteries and needs to be urgently addressed. Very recently, two examples of engineered high-mass loading electrodes with redox polymers have been shown. On the one hand, Marcilla et al. proposed the use of anthraquinone-based conjugated microporous polymers in the presence of carbon nanostructures and further processing into self-supported buckypaper electrodes.^71^ This effective method enables high-mass-loading hybrid electrodes (up to 60 mg cm^–2^) with low carbon content (20 wt %), which attained high gravimetric capacity (83.7 mAh g_electrode_^–1^), high areal capacity (6.3 mAh cm^–2^), good rate capability (0.8 mAh cm^–2^ at 10 C), and remarkable cycle stability (>80% capacity retention over 1000 cycles). On the other hand, Esser et al. investigated the influence of the carbon type in poly(3-vinyl-*N*-methylphenothiazine) electrodes. Compared to the state-of-the-art conductive carbon Super C65 employed in many organic battery electrodes, Ketjenblack EC-300J and EC-600J resulted in a 3D structure of the electrode. The studies demonstrate that a dense packing of the carbon particles in the electrode is decisive for the stable immobilization of PVMPT while maintaining its long-term cycling performance.^72^

A possibility offered by its synthetic versatility is the development of polymers that show several redox groups in the same polymer backbone. This allows that the same polymer material could be used as an anode and cathode simultaneously in a symmetric organic battery configuration. This fact facilitates the electrode processing and the preparation of simple organic batteries as recently shown by some of us in the case of a polyimide including a phenazine-containing monomer.^73^

Redox polymers are also known for the versatility of their redox chemistry and the possibility of using different ions from Li and Na including multivalent ions such as Mg, Ca, or Al to organics. For instance, several redox polymers including catechol, polyimides, or phenothiazine have recently been demonstrated as universal hosts for aqueous rechargeable batteries based on mono-, di-, or trivalent cations.^74−76^ As two seminal examples of the possibilities of using multivalent ions, an ultrahigh performance zinc–organic battery using a poly(catechol) cathode in concentrated aqueous electrolytes was recently shown by Marcilla and co-workers,^77^ and Esser et al. demonstrated recently an aluminum–organic battery.^78^

The design of redox polymers that are biobased or biodegradable is an important research direction. As a seminal example, Lutkenaus and Wooley designed TEMPO and viologen functional polymers including a biodegradable polypeptide backbone to be used in organic radical batteries.^79^ These redox-active polypeptides perform as active materials that are stable during battery operation and subsequently degrade to generate amino acids. In another example, Inganas et al. proposed the use of lignin as electrode material for supercapacitors in combination with conducting polymers.^80^ Liedel and Antonietti investigated the use of polyphenol natural molecules such as dopamine to develop polymer cathodes with high charge storage for batteries.^81^

Another important research direction is the use of redox polymers for the development of new battery technologies such as organic redox flow batteries (RFBs) and polymer–air or full organic printable batteries. Redox polymers are being proposed for the replacement of the problematic vanadium compounds, currently used as active material in RFBs. In recent years, TEMPO, viologen, and quinone molecules have proven to be the most suitable redox small organic molecules for application in aqueous RFBs due to a good long-term cycling stability (>1000 charge/discharge cycles), high Coulombic efficiencies (>98%), and energy densities up to 10 Wh/L. The main advantage of using redox polymers instead of redox-active small organic molecules in RBFs is that, in principle, due to the big size of macromolecules it is possible to substitute the inefficient and expensive ion-selective membrane, necessary to keep the two electrolytes separated avoiding crossover or cross-contamination, by cheaper size-exclusion membranes that still allow small ions to pass as charge carriers.^82^ The main disadvantage of the use of polymers is that the viscosity of the redox electrolytes significantly increases with the concentration of polymer, which eventually causes a serious penalty in the electrolyte transport properties and a relevant increase in pumping electrolyte cost. In this sense, an interesting approach is to use the redox-active compounds not dissolved in solution but in the form of micelles, colloids, or microparticles. For instance, Schubert et al. recently showed the development of TEMPO containing microparticles by emulsion polymerization.^83^

**Figure 5:**
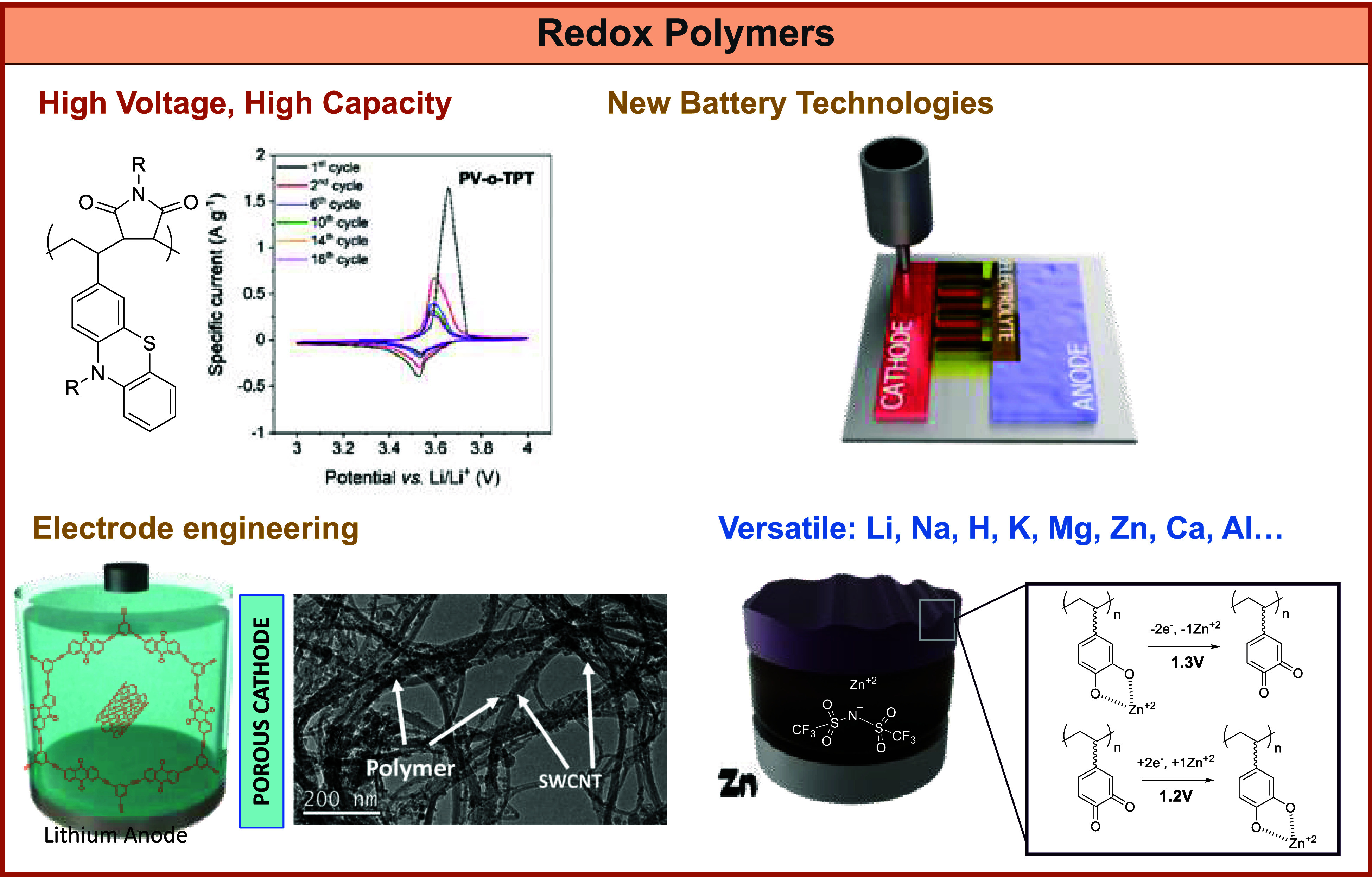
Representation of redox polymers investigated as battery electrode active materials.

## 4 Future Directions

Today the race is open worldwide to develop next-generation batteries that improve the performance of current batteries. Polymers will play an important role in this race in particular to overcome issues such as raw materials availability, safety, low weight, printability, and flexibility. The perspective is that new polymers will be developed in the next years following the needs indicated in the previous sections for polymer binders, porous separators, polymer electrolytes, or redox polymers.

Furthermore, there are aspects that are common to all the polymers used in the different parts of the battery. One of them is sustainability, both with respect to sourcing active materials and for overall recyclability of the battery. This topic will become more important in the future due to the massive production of batteries in the gigafactories. Aspects like sustainability and biodegradability of the polymers used in the battery in addition to their processability in green solvents such as water are becoming important. Here, the use of new biobased polymers, biopolymers, and polymers coming from polymer recycling and upcycling of plastic waste should become important in the next few years.^84,85^

A second important aspect is related to battery production technologies. Current wet processing methods of batteries involve the use of organic solvents (NMP, etc.) toxic for humans and the environment overall. Here, a sustainable alternative would be “dry” electrode fabrication through the use of a fast extrusion process, which is already well-known in the plastics industry. In this case, the active materials, electrolytes, polymer binders, and carbon additives can be physically mixed in an extruder, and the powder mixtures are subsequently subjected to lamination. This production process is becoming very attractive and important for future battery manufacturing. Thus, there will also be an opportunity for polymer materials design, specifically for these new manufacturing processes.

Current battery electrode fabrication is based on well-known 1D or 2D roll-to-roll processes. In the past years, we are experiencing a fast development of additive manufacturing methods including new 3D and 4D printing technologies. Many opportunities are seen in the use of additive manufacturing in batteries, including the development of 3D printing electrode designs in the module architectures and battery configurations. Besides, the batteries could have customized shapes, which could change how batteries are integrated into the product design. Here, functional polymers for polymer electrolytes, advanced printing technologies, and new device designs need to be developed to take full advantage of all the opportunities offered by additive manufacturing for batteries.^86,87^

The third aspect is the opportunity offered by the megatrend and recent developments in artificial intelligence and machine learning for materials discovery. However, the area is still in its infancy due to the complexity of applying AI models to polymers, insufficient prediction accuracy of material properties, and large exploration space for candidate structures. Some works have already shown the possibilities of using AI for polymer electrolytes. In the past year, different models such as a chemistry-informed neural network^88^ and a data trend analysis system for materials using quantum-inspired annealing^89^ have been applied to accurately predict ionic conductivity in solid polymer electrolytes. Using the trained model, ionic conductivity values were predicted for several thousand candidate solid polymer electrolytes. Also very recently, an AI-driven and machine learning framework was developed for discovery of organic battery cathode materials.^90^ This area is expected to grow in the next few years and expand the palette of polymers available for batteries.
